# Correction: Path analysis based on genetic association of yield components and insects pest in upland cotton varieties

**DOI:** 10.1371/journal.pone.0272390

**Published:** 2022-07-27

**Authors:** Mussarat Shaheen, Hafiz Abdul Rauf, Muhammad Ahmed Taj, Muhammad Yousaf Ali, Muhammad Amjad Bashir, Sagheer Atta, Hasnain Farooq, Reem A. Alajmi, Mohamed Hashem, Saad Alamri

The affiliation for the eighth author is incorrect. Saad Alamri is not affiliated with #9 but with #8: Department of Biology, College of Science, King Khalid University, Abha, Saudi Arabia,

## References

[1] Shaheen M, Abdul Rauf H, Taj MA, Yousaf Ali M, Bashir MA, Atta S, et al. (2021) Path analysis based on genetic association of yield components and insects pest in upland cotton varieties. PLoS ONE 16(12): e0260971. 10.1371/journal.pone.0260971 34969047 PMC8717984

